# Transcriptional regulation of HSPB1 by Friend leukemia integration-1 factor modulates radiation and temozolomide resistance in glioblastoma

**DOI:** 10.18632/oncotarget.27425

**Published:** 2020-03-31

**Authors:** Yetirajam Rajesh, Angana Biswas, Payel Banik, Ipsita Pal, Subhayan Das, Sachin A. Borkar, Hardik Sardana, Abhijit Saha, Swadesh K. Das, Luni Emdad, Paul B. Fisher, Mahitosh Mandal

**Affiliations:** ^1^School of Medical Science and Technology, Indian Institute of Technology Kharagpur, Kharagpur, India; ^2^Department of Neurosurgery & Gamma Knife, All India Institute of Medical Sciences, New Delhi, India; ^3^Radiation Department, UGC DAE Consortium, Jadavpur University, Kolkata, India; ^4^Department of Human and Molecular Genetics, Virginia Commonwealth University, School of Medicine, Richmond, VA, USA; ^5^VCU Institute of Molecular Medicine, Virginia Commonwealth University, School of Medicine, Richmond, VA, USA; ^6^VCU Massey Cancer Center, Virginia Commonwealth University, School of Medicine, Richmond, VA, USA

**Keywords:** HSPB1, RNAseq, Fli-1, radioresistant GBM, temozolomide resistant GBM

## Abstract

Glioblastoma (GBM) is the most common primary brain tumor and is invariably fatal. Heat shock proteins (HSPs) provide protein signatures/biomarkers for GBM that afford potential as targets for developing anti-GBM drugs. In GBM, elevated expression of hypoxia inducible factors under the influence of Ets family proteins significantly promotes the expression of HSPs. RNAseq analysis identified HSPB1 as a prominent upregulated HSP in GBM and in radiation resistant/temozolomide resistant (radio/TMZR) GBM. Here, we established friend leukemia integration 1 (Fli-1), a member of Ets family to be playing a transcriptional regulatory role on the HSPB1 gene. Fli-1 binds to nucleotide residues GGAA at binding sites 3, 6 and 7 in the 5-kb upstream region of HSPB1. Fli-1 has been linked to oncogenic transformation with upregulation in radio/TMZR GBM. Overexpression of Fli-1 in GBM promotes resistance, whereas Fli-1 knockdown in radio/TMZR GBM cells suppresses resistance. We identify the underlying molecular mechanisms of Fli-1-mediated regulation of HSPB1 that drive extracellular matrix remodeling and epithelial to mesenchymal transition in radio/TMZR GBM cells. This study uncovers Fli-1 as a potential therapeutic target for combating radiation and temozolomide resistance in GBM.

## INTRODUCTION

GBM account for the majority of brain tumors with high proliferative potential, infiltrative (invasive) growth behavior, intratumoral heterogeneity and tumor recurrence. Rapid proliferation and invasion by GBM cells are major pathologic features contributing to failure of conventional therapeutic regimens [1, 2]. Conventional “gold-standard” therapies that employ radiation and temozolomide (chemotherapy) have proven unsuccessful in curing this aggressive cancer and frequently promote acquired therapeutic resistance following disease recurrence. Defining the underpinnings of GBM development and progression has potential to provide a path forward in creating effective GBM therapies [1, 3]. Omics studies in search of protein signatures and biomarkers for GBM specifically highlight upregulation of heat shock proteins (HSPs) in GBM [1, 4]. The HSPs promote tumor growth by stimulating cell proliferation and inhibiting cell death pathways [4]. They also display chaperone activity for many proteins including matrix degrading enzymes involved in extracellular matrix (ECM) degradation. HSPs coordinate multiple components of the ECM remodeling system revealing a positive correlation between high expression of HSPs and invasive capacity of GBM cells. HSPs also play an essential extracellular role during the infiltrative phase in GBM through binding to MMPs [5]. A potential role for HSPs in facilitating epithelial-mesenchymal transition (EMT) in cancer has also been reported [4]. HSP up regulation is evident in gliomagenesis as well as in acquisition of radio- (radiation) and temozolomide- (TMZ) resistance (radio/TMZR) [4]. Hence, HSPs represent prospective targets to develop effective clinical strategies for advancing rational anti-GBM drug development.

HSPB1 was identified using RNA sequencing amplification (RNAseq) as the most upregulated heat shock family protein in GBM and in radio/TMZR GBM. For that reason, we focused on defining the transcriptional regulator(s) of HSPB1 to understand the underlying mechanism controlling GBM progression and acquisition of radio/TMZR. GBM cells have elevated expression of hypoxia inducible factors (HIFs) and transcription of hypoxia-related genes, and intratumoral EMT [6]. Moreover, a significant role of hypoxia in elevating the expression of several HSPs in various tissues is well documented [7–9]. The ETS family of transcription factors direct the expression of oncogenes and tumor suppressor genes, and regulate blood vessel formation, invasion, and metastasis genes, which correlate with poor patient survival [10–13]. In total, a preponderance of evidence suggests a distinct link between HSPB1 and the Ets family of transcription factors [9, 11, 13].

Through computational and biological studies the Friend leukemia integration 1 transcription factor (Fli-1) was identified in the 5-kb upstream region of the HSPB1 gene. We presently validate a critical role of Fli-1 in regulating HSPB1 in patient-derived GBM. Fli-1 is a member of the ETS transcription factor family, a target of insertional activation by Friend murine leukemia virus (F-MuLV) and is preferentially expressed in vascular endothelial cells and hematopoietic tissues [14]. It affects cellular proliferation and tumorigenesis in Ewing sarcoma and primitive neuroectodermal tumors and also plays a crucial role in normal development, hematopoiesis, and oncogenesis [11, 12, 15–18]. Fli-1 overexpression has been established as a biomarker in melanoma [19], ovarian cancer [20], endometrial cancer [21], breast cancer [22], and nasopharyngeal carcinoma [23]. However, no previous studies have identified a correlation between Fli-1 protein and HSPB1 or its association with radio/TMZR in GBM. Fli-1 overexpression promotes radiation- and TMZ-resistance in GBM cells resulting in morphological and molecular characteristics in these cells that are similar to those of radio/TMZR GBM cells. Conversely, Fli-1 knockdown in radio/TMZR GBM cells sensitizes these cells to radiation and TMZ concomitantly promoting morphological and molecular characteristics similar to those of parental GBM cells. In total, these studies provide compelling evidence that Fli-1 can directly regulate radiation and temozolomide resistance in GBM and targeting Fli-1 has potential as an effective therapy for treating both primary GBM and radio/TMZR GBM.

## RESULTS

### Expression of different HSP family proteins in GBM

**Figure 1:**
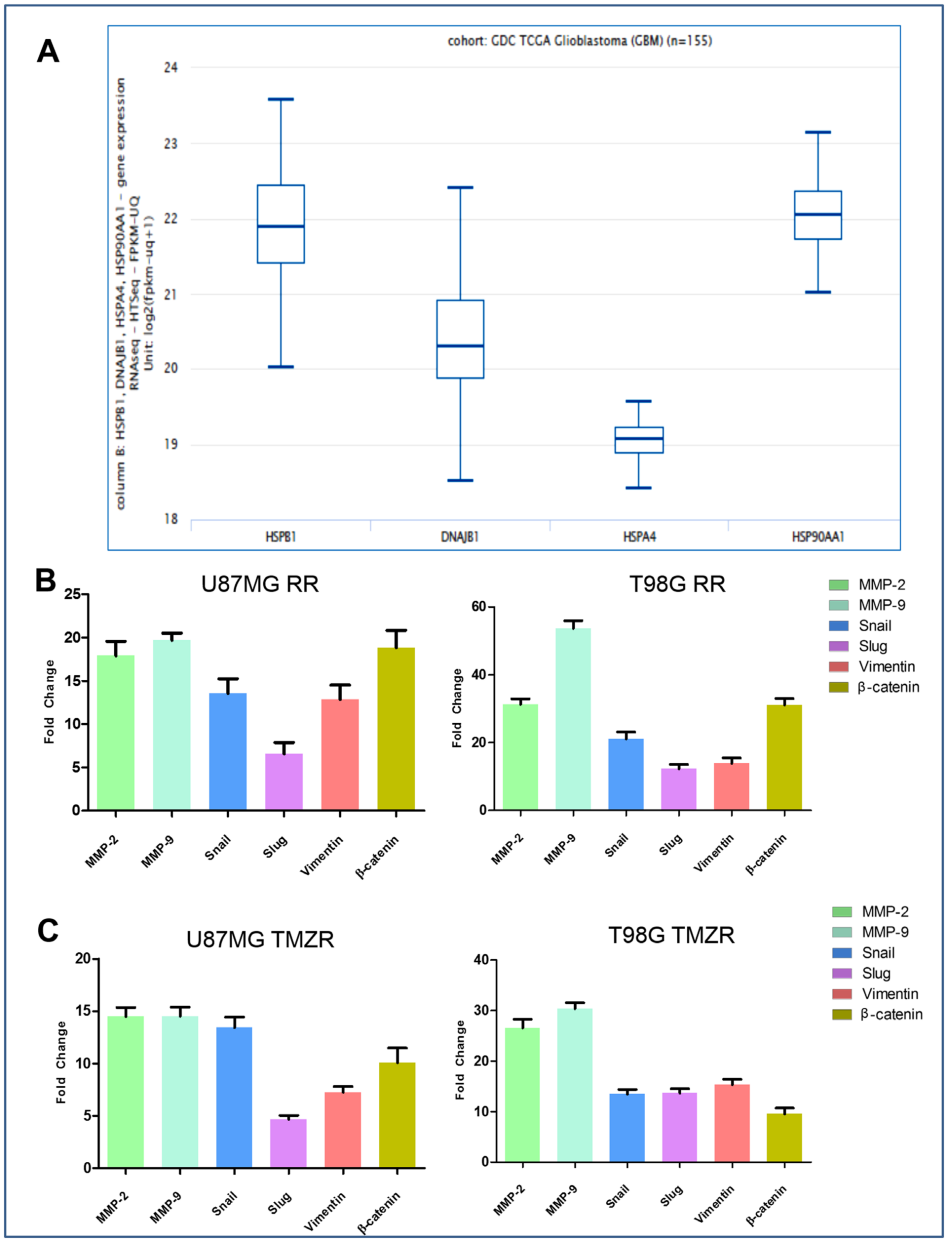
Comparative expression pattern of different HSPs and determination of radiation resistance and TMZ resistance (radio/TMZR) in GBM. (**A**) Graphical representation of absolute RNAseq quantification of different HSPs involved in GBM from TCGA cBioPortal (n = 155). (**B**) Relative expression (RT-qPCR; average ± S. E.) of MMP-2, MMP-9 and EMT markers of U87MG RR and T98G RR cells. (**C**) Relative expression (RT-qPCR; average ± S. E.) of MMP-2, MMP-9 and EMT markers of U87MG TMZR and T98G TMZR cells. Normalization of data was performed with 18S rRNA. Data representation is with respect to expression of respective genes in U87MG RR/TMZR and T98G RR/TMZR cells vs. expression in U87MG and T98G cells, respectively.

Using RNAseq quantification of different HSPs in GBMs obtained from the GDC TCGA glioblastoma cohort (n = 155) cbioportal (Figure 1A) we identified expression of HSPB1 to be highest in GBM.

### Generation and characterization of radio/TMZ-resistant (radio/TMZR) GBM cells

**Figure 2:**
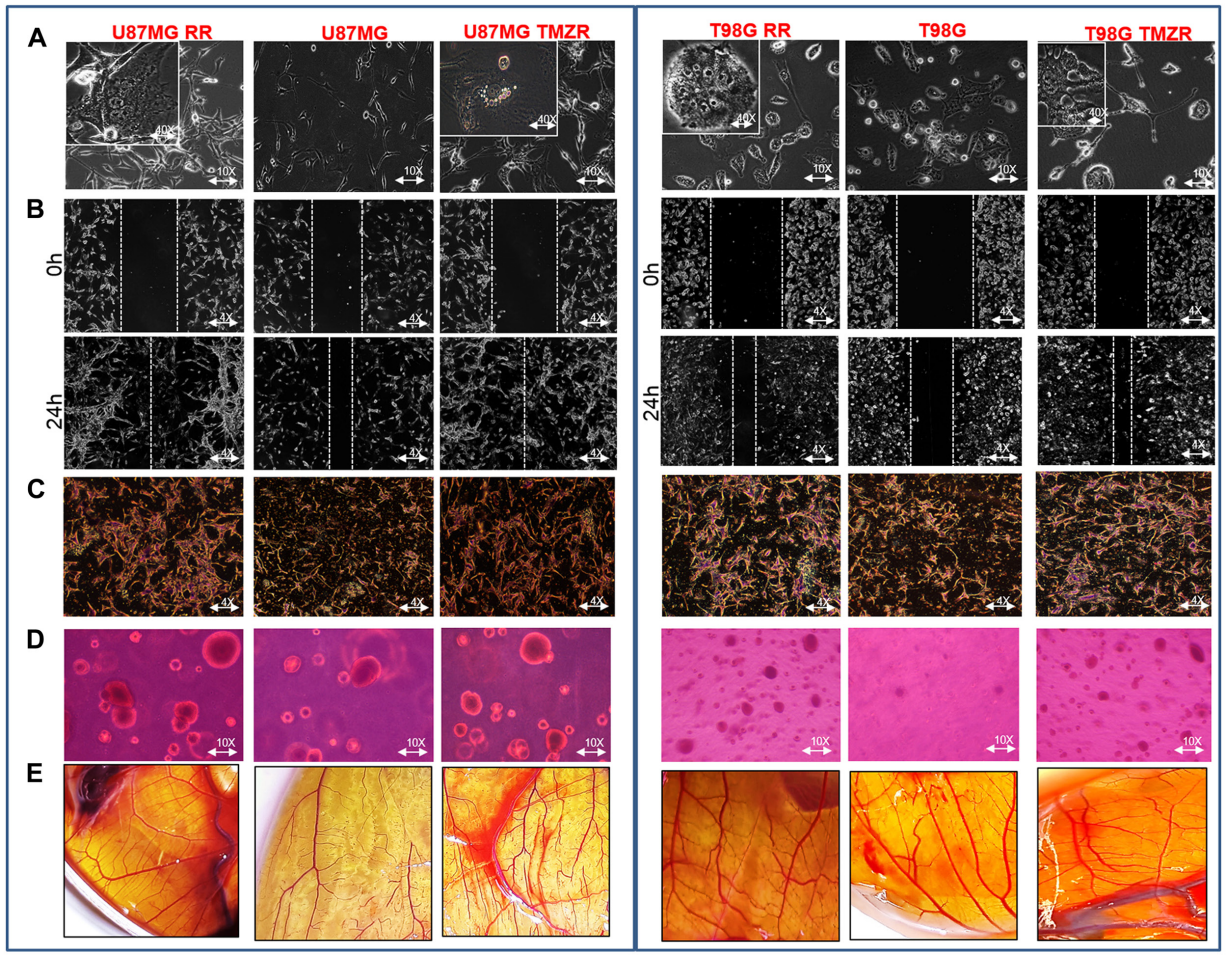
Characterization of radiation-resistant and TMZ-resistant (RR/TMZR) GBM cells. (**A**) Phase contrast microscopy images of U87MG RR/TMZR and T98G RR/TMZR cells displaying long cellular extensions (EMT-like traits) (10× magnification) and multi-nucleation in resistant cells (40× magnification). (**B–E**) Wound-healing, Boyden-chamber, soft-agar and CAM assays evaluating migratory, invasive, anchorage independent growth and angiogenesis potential, respectively, of U87MG, U87MG RR, U87MG TMZR, T98G, T98G RR and T98 TMZR cells.

U87MG and T98G cells were irradiated with 2 Gy/day (total of 30 Gy; 5× per week for 3 wks) using a GR-12 irradiator (^60^Co γ rays, dose rate- 6.85kGy/h) (details in Methods). Survival of parental and putative radio-resistant cells was determined following irradiation confirming acquisition of radiation resistance in U87MG RR and T98G RR GBM vs. parental GBM cells (Supplementary Figure 1A). The radio-resistant GBM cells (Figure 1B) exhibited transcriptional upregulation in EMT markers (β-Catenin, Vimentin, Snail, Slug) and ECM remodeling proteins (MMP-2 and MMP-9). Characteristics of the mesenchymal phenotype, *i.e.*, spindle-like morphology, loss of cell-to-cell contact, increased cell scattering and multi-nucleation, were observed in U87MG RR and T98G RR cells (Figure 2A). The radio-resistant cells showed high migratory ability (Figure 2B), invasive potential (Figure 2C), anchorage-independent growth (Figure 2D) and angiogenesis (Figure 2E).

TMZ-resistant cell lines (U87MG TMZR and T98G TMZR) were generated by stepwise and continuous exposure of the parental U87MG and T98G cells to increasing concentrations of TMZ over a 10–12 month period [24]. The U87MG and T98G cells were treated with a sub-lethal dose of TMZ (50 µM). The proliferative sensitivity of TMZ in U87MG and T98G was determined by conventional MTT assay. In parental U87MG and T98G cells, the IC_50_ value of TMZ was 392.2 µM and 717.2 µM, respectively; in TMZR cells the IC_50_ value of TMZ was 1557.3 µM and 2196.3 µM, respectively. These results suggested that these GBM cells have developed TMZR (Supplementary Figure 1B). They also exhibited transcriptional upregulation of EMT markers (β-Catenin, Vimentin, Snail, Slug) and ECM remodeling proteins (MMP-2 and MMP-9) (Figure 1C). Characteristics of the mesenchymal phenotype, *i.e.*, spindle-like morphology, loss of cell-to-cell contact and increase in cell scattering and multi-nucleation were observed in U87MG TMZR and T98G TMZR cells (Figure 2A). The TMZ-resistant cells showed high migratory potential (Figure 2B), invasive properties (Figure 2C), anchorage independent growth (Figure 2D) and angiogenesis (Figure 2E).

**Figure 3:**
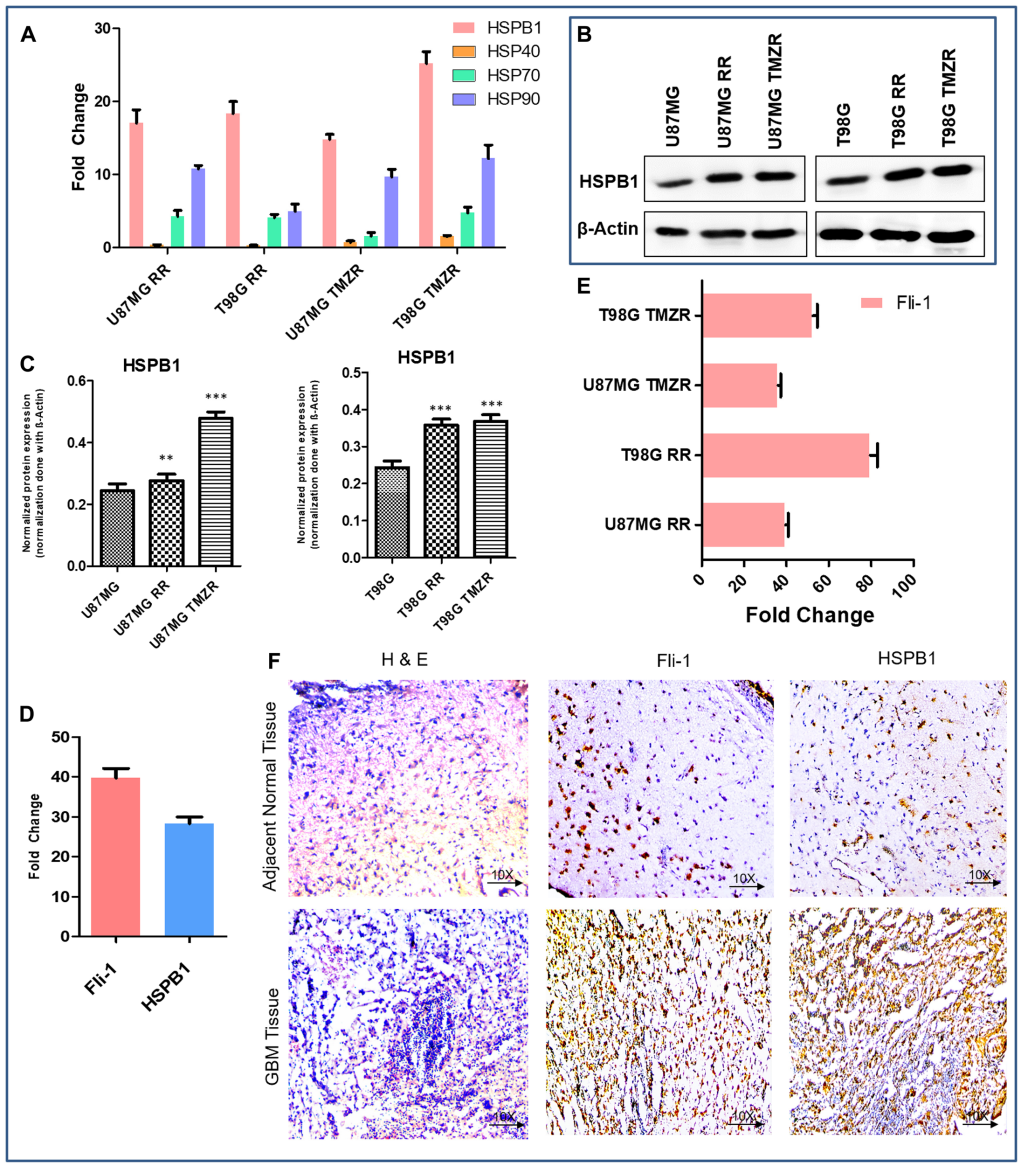
Comparative expression pattern of different HSPs and Fli-1 in GBM. (**A**) Relative expression of different HSPs involved in radiation-resistance and TMZ-resistance (RR/TMZR) in GBM cells. Normalization of data is performed with 18S rRNA. (**B**) Western blotting analysis of HSPB1 in U87MG, U87MG-RR, U87MG-TMZR and T98G, T98G-RR, T98G-TMZR cells. During contrast adjustments of certain blots in panel b, background removal occurred. (**C**) Densitometry plot of (B). Each bar represents the average of three independent experiments. The level of significance is indicated by ^*^ *P* < 0.05, ^**^ *P* < 0.01, ^***^ *P* < 0.001. (**D**) Relative expression of Fli-1 and HSPB1 in GBM tissue samples presented as fold-change vs. normal adjacent tissue. (**E**) Relative expression of Fli-1 involved in radiation-resistant and TMZ resistant (RR/TMZR) GBM cells. (**F**) Immunohistochemistry of human adjacent normal and GBM tissue sections. Hematoxylin and Eosin staining and expression of Fli-1 and HSPB1.

The relative expression of HSP members in GBM were analyzed by RT-qPCR in radio/TMZ-resistant GBM cells U87MG, U87MG RR, U87MG TMZR and T98G, T98G RR, T98G TMZR (Figure 3A). Among the HSPs, the transcriptional level of HSPB1 was found to be significantly upregulated in both radiation-resistant and TMZ-resistant GBM cells in comparison to parental GBM cells. The translational protein levels of HSPB1 was also significantly upregulated in radio/TMZ-resistant GBM cells in comparison to parental GBM cells (Figure 3B, 3C).

### Fli-1 is a predicted transcription factor in the upstream region of HSPB1

**Figure 4:**
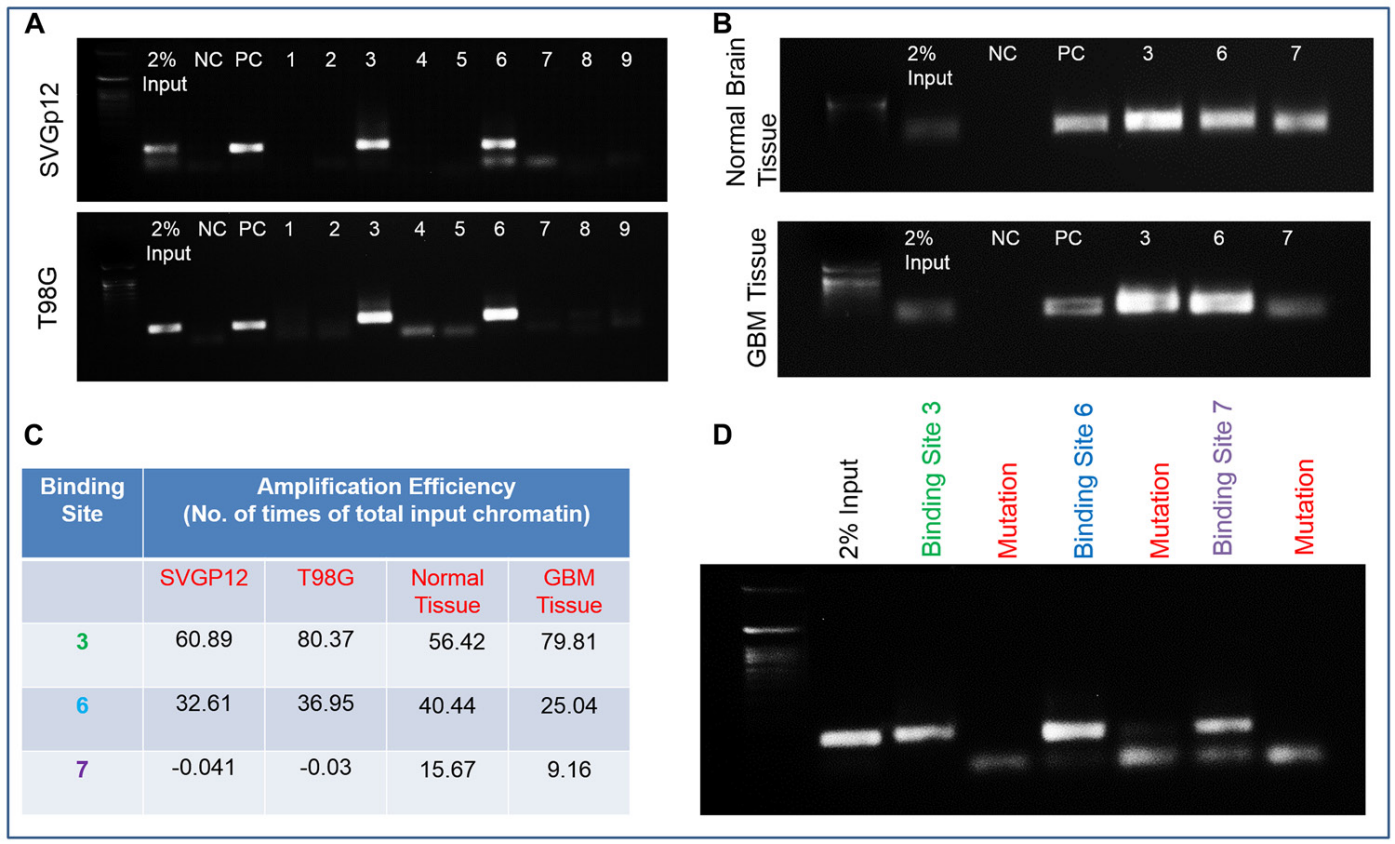
Fli-1, a transcription factor in the upstream region of HSPB1. (**A**) Quantification of ChIP DNA by standard PCR of nine predicted binding sites in chromatin from SVGP12 and T98G cells. (**B**) Quantification of ChIP DNA by standard PCR of selected binding sites (primer no. 3, 6 and 7) in chromatin from human adjacent normal and GBM tissues. (**C**) Quantitative Real-Time PCR of selected binding sites (primer no. 3, 6 and 7) in SVGP12 and T98G cells; human adjacent normal and GBM tissue. (**D**) RT-PCR image showing no amplification of ChIP DNA after mutation of binding sites 3, 6 and 7 with their respective controls.

Using ALIBABA and PATCH software, the 5-kb upstream region of HSPB1 (Supplementary Figure 2) was predicted to contain the transcription factor Fli-1 (Supplementary Figure 3). Computational and manual prediction detected 9 probable binding sites for transcription factors in the 5-kb upstream region of HSPB1 (Supplementary Figure 2). Both Fli-1 and HSPB1 expression were found to be elevated in human GBM tissue samples in comparison to adjacent normal tissue (Figure 3D). Quantitative PCR demonstrated upregulated expression of Fli-1 in radio/TMZR GBM cells (Figure 3E). Additionally, IHC of human GBM tissue samples indicated higher expression of Fli-1 and HSPB1 in comparison to adjacent normal tissue samples (Figure 3F). In addition, the quantification of ChIP DNA by standard PCR and quantitative real time PCR of nine predicted binding sites in chromatin from SVGP12 and T98G cells confirmed the binding sites 3, 6, and 7 in the 5-kb upstream of HSPB1 bound to Fli-1 (Figure 4A, 4C). Binding of Fli-1 was also clinically validated in chromatin from human adjacent normal and GBM tissues by quantification of ChIP DNA using standard PCR of selected binding sites (primers no. 3, 6 and 7) (Figure 4B, 4C).

### Fli-1 binds to GGAA in the 5-kb upstream of HSPB1

The confirmation of binding of Fli-1 to binding sites 3, 6 and 7 in the 5-kb upstream region of HSPB1 was provided by mutating the predicted binding sites, thereby functionality eliminating binding. The entire length of this oligonucleotide (~120-bp) was then synthesized to ensure that there was sufficient length for binding with the Fli-1 transcription factor. The mutated site was present in the mid-region of the synthesized oligonucleotide. The oligonucleotide was then incubated along with the transcription factor Fli-1 to monitor *in vitro* binding. ChIP analysis was then done with the synthesized oligonucleotide-protein complex in place of cell chromatin to mimic standard ChIP conditions. No amplification band was observed when using the mutated oligonucleotide. However, a clear amplification band was evident when the experiment was performed with control (non-mutated) oligonucleotides. This proves that 3, 6 and 7 are true binding sites for Fli-1 in the upstream region of HSPB1 (Supplementary Figure 4; Figure 4D).

### Fli-1 regulates radiation- and TMZ-resistance in GBM cells

**Figure 5:**
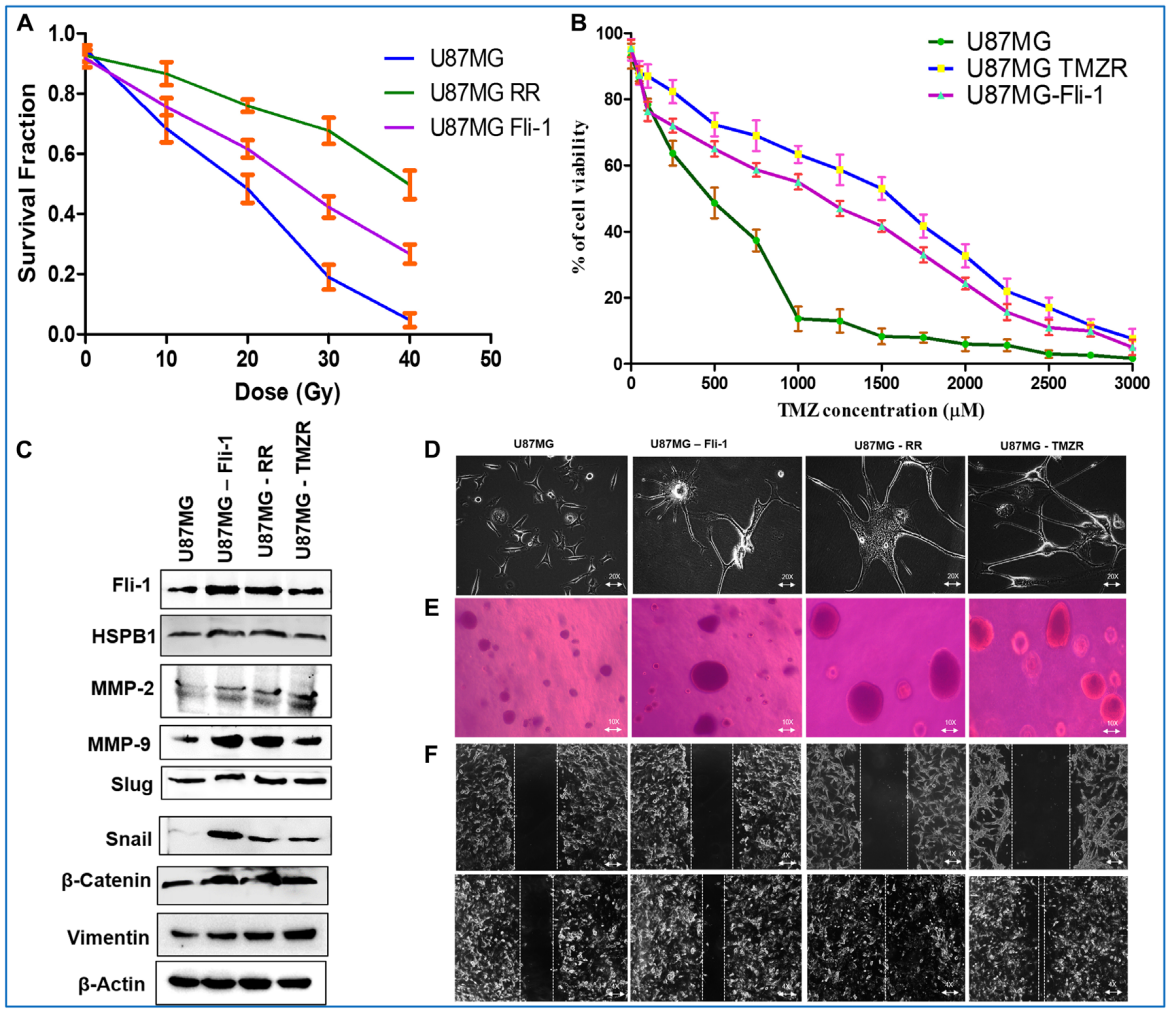
Fli-1 overexpressing stable U87MG cells displaying radio/TMZ resistance-like characteristics. (**A**, **B**) Determination of survival fraction and IC_50_ of radiation and TMZ in parental U87MG cells, radiation-resistant U87MG RR, TMZ-resistant U87MG and U87MG Fli-1 cells. (**C**) Western blotting analysis of Fli-1, HSPB1, MMP-2, MMP-9 and EMT proteins in U87MG, U87MG RR, U87MG TMZR and U87MG Fli-1 cells. Densitometry of (C) is in Supplementary Figure 6. During contrast adjustments of certain blots in panel c, background removal occurred. (**D**) Phase contrast microscopy images of U87MG RR/TMZR and U87MG Fli-1 cells. U87MG Fli-1 cells showing long cellular extensions (EMT-like traits) comparable to resistant cells. (**E**) Soft-Agar assays evaluating anchorage independent growth of U87MG, U87MG RR, U87MG TMZR, U87MG Fli-1 cells. (**F**) Wound-healing assays evaluating migratory potential of U87MG, U87MG RR, U87MG TMZR, U87MG Fli-1 cells.

A Fli-1 overexpression plasmid was constructed by amplifying the Fli-1 gene from the T98G cell line using sequence-specific primers. The Fli-1 plasmid was cloned into a TA vector, sequenced to check accuracy of the amplified gene and the intact gene along with its respective start and stop codons (present in the sequence) (Supplementary Figure 5) was cloned into the pEGFP mammalian expression vector using appropriate restriction enzymes as described [5]. This plasmid construct was used to overexpress Fli-1 in U87MG GBM cells (U87MG Fli-1). U87MG Fli-1 cells exhibited an intermediate radiation survival phenotype as compared to U87MG and U87MG RR, *i.e.*, higher resistance to radiation than U87MG, but lower radiation resistance than U87MG RR (Figure 5A). U87MG Fli-1 has an IC_50_ of 1179.2 µM TMZ in comparison to an IC_50_ of 397.2 µM TMZ for U87MG and 1561.3 µM TMZ for U87MG TMZR (Figure 5B). The Fli-1, HSPB1, MMP-2, MMP-9 and EMT proteins (Slug, Snail, β-Catenin and Vimentin) were upregulated in U87MG Fli-1 cells displaying similar molecular characteristics as U87MG RR and U87MG TMZR cells (Figure 5C; Supplementary Figure 6). Phase contrast microscopy images of U87MG Fli-1 cells showed long cellular extensions (EMT-like traits) similar to resistant cells (U87MG RR/TMZR) (Figure 5D). Soft-Agar assays indicated enhanced anchorage independent growth potential of U87MG Fli-1 cells vs. U87MG parental cells that were comparable to U87MG RR and U87MG TMZR cells (Figure 5E). Wound-healing assays confirmed higher migratory potential of U87MG Fli-1 cells vs. U87MG parental cells that were comparable to U87MG RR and U87MG TMZR cells (Figure 5F). In contrast, no significant differences in any of the above mentioned properties were evident in U87MG cells transfected with an empty vector (data not shown).

**Figure 6:**
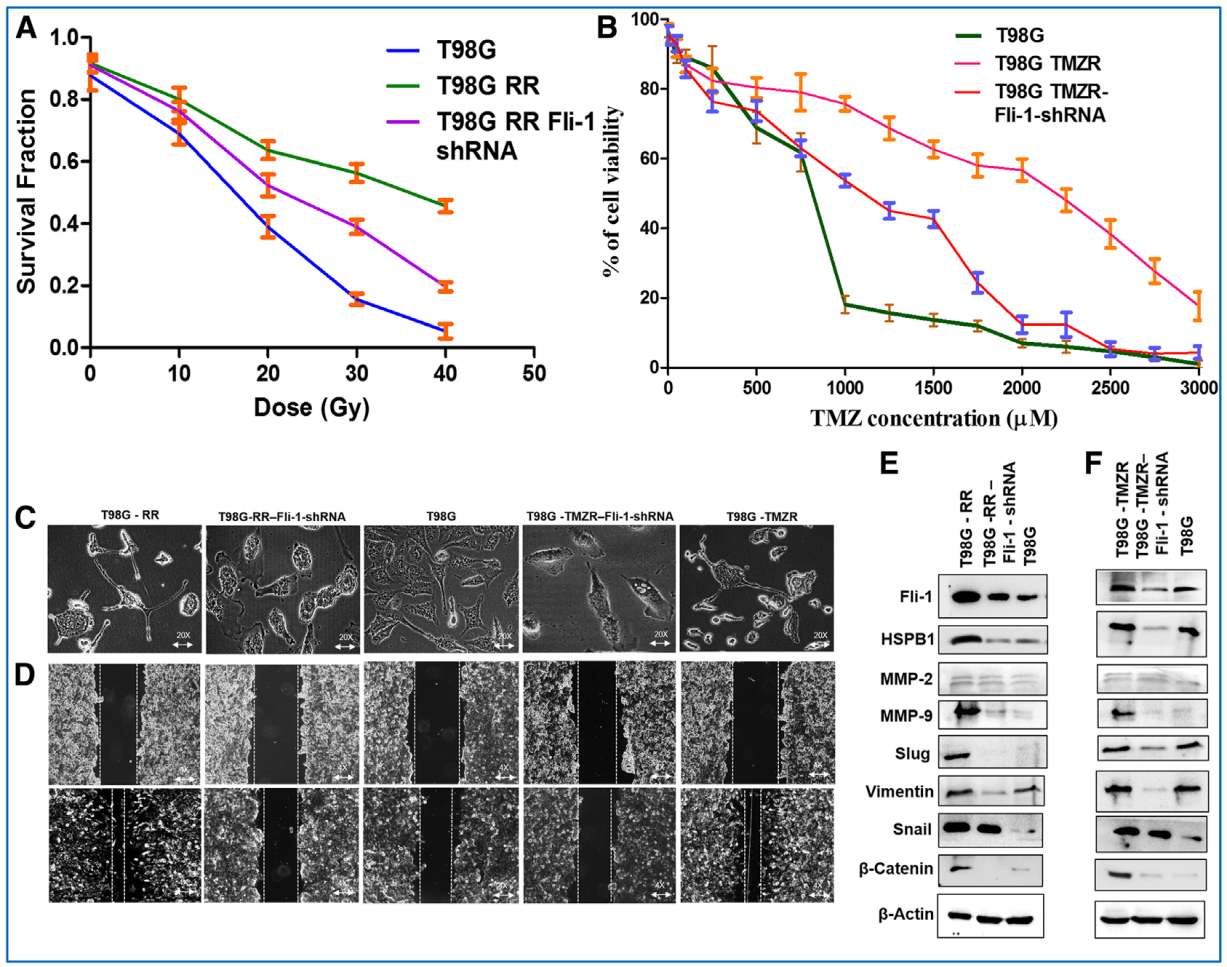
Therapeutic resistance is reversed in Fli-1 knockdown stable T98G RR Fli-1 shRNA and T98G TMZR Fli-1 shRNA cells. (**A**, **B**) Determination of survival fraction and IC_50_ of radiation and TMZ in parental T98G, radiation-resistant T98G RR, TMZ-resistant T98G TMZR, T98G RR Fli-1 shRNA and T98G TMZR Fli-1 shRNA cells. (**C**) Phase contrast microscopy images of parental T98G, radiation-resistant T98G RR, TMZ-resistant T98G TMZR, T98G RR Fli-1 shRNA and T98G TMZR Fli-1 shRNA cells. T98G RR Fli-1 shRNA and T98G TMZR Fli-1 shRNA cells showing absence of long cellular extensions (EMT-like traits) similar to T98G cells. (**D**) Wound-healing assay evaluating migratory potential of parental T98G, radiation-resistant T98G RR, TMZ-resistant T98G TMZR, T98G RR Fli-1 shRNA and T98G TMZR Fli-1 shRNA cells. (**E**, **F**) Western blotting analysis of Fli-1, HSPB1, MMP-2, MMP-9 and EMT proteins in parental T98G, radiation-resistant T98G RR, TMZ-resistant T98G TMZR, T98G RR Fli-1 shRNA and T98G TMZR Fli-1 shRNA cells. Densitometry plot of (E, F) is in Supplementary Figure 7A and 7B. During contrast adjustments of certain blots in panels e and f, background removal occurred.

As a further proof of the role of Fli-1 in defining both radiation- and TMZ-resistance in GBM cells, Fli-1 expression was knocked down using Fli-1 shRNA in T98G RR and T98G TMZR cells. The IC_50_ of TMZ resistance in T98G is 2273.1 µM and of T98G TMZR Fli-1 shRNA is 1092.3 µM that is comparable to T98G (732.6 µM) cells (Figure 6A, 6B). Phase contrast microscopy images of T98G RR Fli-1 shRNA and T98G TMZR Fli-1 shRNA cells did not show long cellular extensions (EMT-like traits), which is similar to parental T98G cells (Figure 6C). Wound-healing assays demonstrated reduced migratory potential of T98G RR Fli-1 shRNA and T98G TMZR Fli-1 shRNA cells vs. T98G cells (Figure 6D). Western blotting analysis of Fli-1, HSPB1, MMP-2, MMP-9 and EMT proteins in T98G RR Fli-1 shRNA and T98G TMZR Fli-1 shRNA cells indicated downregulation in expression of these proteins resulting in comparable levels as observed in T98G parental cells (Figure 6E, 6F; Supplementary Figure 7A, 7B). Transfection with control shRNA exerted no significant effect on T98G cells (data not shown). Collectively, these data provide evidence for a direct relationship between Fli-1 expression in GBM and resistance to radiation and TMZ.

## DISCUSSION

Glioblastoma multiforme (GBM) accounts for the majority of brain tumors that portend poor patient prognosis, despite conventional therapeutic strategies including surgery, radiation and/or chemotherapy [1]. By elucidating the molecular pathways involved in gliomagenesis, it could be possible to identify promising GBM-specific therapeutic targets and use these as part of a developmental platform to advance effective therapies for this consistently fatal cancer [2, 3]. Heat shock proteins (HSPs) have been extensively studied and provide a primary protein signature for GBM progression [4]. HSPs serve as a nexus for ECM remodeling and EMT, which are elevated in both acquired radiation and temozolomide resistance in GBM cells [4]. We now confirm upregulation of specific HSPs, particularly HSPB1, in the context of GBM. Moreover, HSPB1 is also markedly upregulated in radiation-resistant and TMZ-resistant (radio/TMZR) GBM cells. Based on these observations, we sought to determine if this specific HSP could provide a viable target for managing major barriers to effective GBM therapy, i.e., acquired radiation resistance and TMZ-resistance. In principle, identifying appropriate oncogenic signaling pathways that regulate gliomagenesis, through transcriptional regulation of HSPB1, is appealing for developing a therapeutic approach to selectively-target therapy-resistant GBM cells. Based on these considerations, we endeavored and succeeded in identifying a potentially relevant transcription factor located in the 5-kb upstream region of the HSPB1 gene, i.e., Fli-1. We then went on to prove that Fli-1 functionally regulates HSPB1 expression.

Overexpression of Fli-1 in GBM cells promoted resistance to both radiation and temozolomide (radio/TMZR), whereas inhibiting Fli-1 expression in GBM cells suppressed these resistance phenotypes. GBM cells exhibit elevated activity of hypoxia inducible factors (HIFs) and transcription of hypoxia-related genes as well as intratumoral EMT [6]. A positive correlation between hypoxia and HSPs is also well documented [7]. The ETS family of transcription factors regulate hypoxia [8] and HSPB1 suggesting that targeting the ETS family of transcription factors might be of therapeutic value [9]. The ETS family of transcription factors are also critical regulators of oncogenes, tumor suppressor genes, and genes related to blood vessel formation, invasion, metastasis, and their expression negatively correlates with patient survival [10–14]. These associations between the ETS family establish a distinct link between HSPB1 and the ETS family.

Data mining identified Friend leukemia integration 1 transcription factor (Fli-1) in the 5-kb upstream region of the HSPB1 gene and corroborated a functional and potential clinical role of Fli-1 regulation of HSPB1 in GBM patients. Fli-1 is a member of the ETS transcription factor family, a target of insertional activation by Friend murine leukemia virus (F-MuLV). It affects cellular proliferation and tumorigenesis in Ewing sarcoma and primitive neuroectodermal tumors and also plays a crucial role in normal development, hematopoiesis, and oncogenesis [11, 12, 15–18]. Fli-1 overexpression in GBM cells recapitulates both the physiological and molecular characteristics of radiation- and TMZ-resistant (radio/TMZR) GBM cells. Similarly, Fli-1 knockdown in radiation-resistant GBM cells results in the reversal of resistance and enhanced sensitization to both radiation and TMZ. These studies support the hypothesis that Fli-1 protein levels regulate HSPB1 gene expression and these changes directly correlate with radio/TMZR in GBM.

The experiments described in our paper establish a positive correlation between Fli-1 expression and radiation- and TMZ-resistance. Fli-1 overexpressing GBM cells exhibited similar characteristics as radio/TMZR GBM cells and knockdown in radiation- and TMZ-resistant GBM cells sensitized these resistant cells to radiation and TMZ treatment and also promoted morphological and molecular characteristics of parental GBM cells. These data support the relevance of developing specific small molecule pharmacological inhibitors of Fli-1 as potentially beneficial therapeutics for GBM and radio/TMZR GBM.

**Figure 7:**
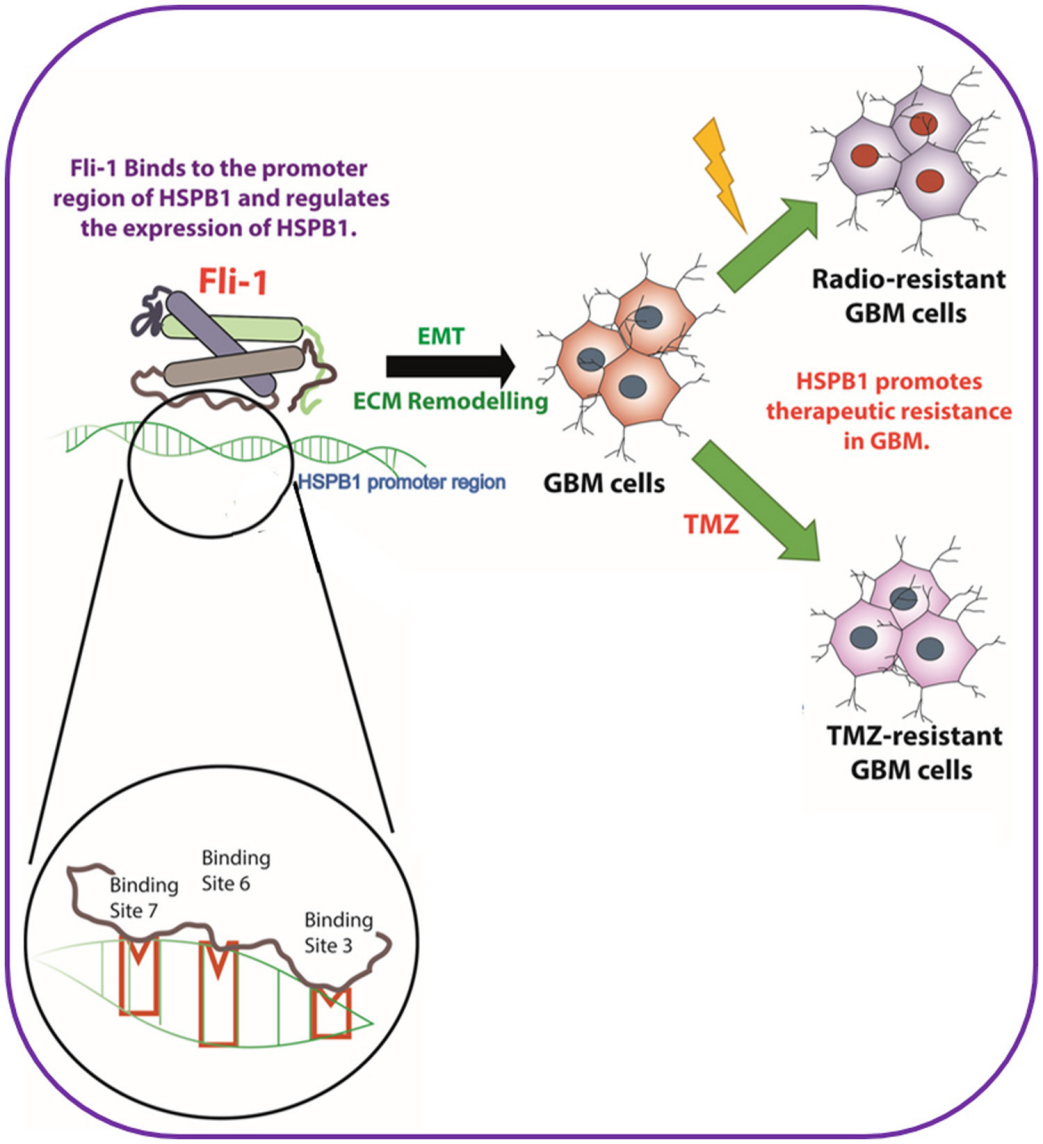
Schematic outline of Fli-1, a transcription factor in the upstream of HSPB1, modulating radiation resistance and temozolomide resistance in glioblastoma.

In conclusion, the current study documents the importance of HSPB1 and Fli-1 in the regulation of radiation- and TMZ-resistance of GBM cells. It illustrates that targeted inhibition of Fli-1/HSPB1-mediates EMT and ECM remodeling signaling axes by genetic (shRNAs/siRNAs) inhibitors can regulate GBM phenotype and might provide novel therapeutic reagents for radio/TMZR glioblastoma (Figure 7).

## MATERIALS AND METHODS

### Human glioblastoma tissue collection and cell cultures

Glioblastoma tumor tissues embedded in paraffin blocks with adjacent normal tissues were collected from GBM patients at the All India Institute of Medical Sciences, India. The institutional human ethical clearance was obtained from All India Institute of Medical Sciences, India – IHEC-LOP/2018/IM0201. The work has been carried out in accordance with the code of ethics of the world medical association. The manuscript has also been prepared in line with the recommendations for the conduct, reporting, editing and publication of scholarly work in medical journals involving humans. An informed consent was obtained for experimentation with human subjects and the privacy rights of human subjects has also been observed. GBM cell lines U87MG, A172 and LN18 were provided by Dr. Ellora Sen, National Brain Research Centre, India; T98G and LN229 were provided by Dr. Annapoorni Rangarajan, Indian Institute of Sciences Bangalore. Cells were grown as described previously [5].

### RNAseq quantification

The cBioPortal (https://www.cbioportal.org/) for analysis of human genome samples was used to determine expression of HSP mRNA in GBM. The TCGA GBM data was analyzed for alterations in HSPs expression in 155 patient samples. We further analyzed the percentage of alterations in GBM [24].

### Generation of radiation-resistant GBM cell lines

Radiation-resistant cell lines (U87MG RR and T98G RR) were generated by exposing U87MG and T98G cells to 2 Gy/day, five times per week for 3 weeks with a total dose of 30 Gy gamma rays. The irradiation was conducted at room temperature using a GR-12 irradiator to expose cells to ^60^Co γ rays (dose rate, 6.85 kGy/hr). Parental U87MG cells were treated similarly except they were sham-irradiated. Both irradiated and sham-irradiated control U87MG cells were sub-cultured every 7 days before the 10th irradiation and were sub-cultured every 10 days after the 10th irradiation. Cells received fresh medium every 2 days. Three weeks after the final irradiation, cell clones were isolated from the irradiated U87MG cell populations and cultured and passaged in the same medium.

### Generation of temozolomide (TMZ)-resistant GBM cell lines

TMZ-resistant cell lines (U87MG TMZR and T98G TMZR) were generated by stepwise and continuous exposure of the parental U87MG and T98G cells to increasing concentrations of TMZ over 10–12 months and were routinely cultured and maintained in their respective media [24].

### Immunohistochemistry

Immunohistochemical analysis and hematoxylin and eosin (H & E) staining of GBM tumor samples and orthotopic GBM tumors grown in mice were performed with different antibodies including Fli-1 and HSPB1 [24].

### Real-Time quantitative PCR (RT-qPCR)

Total cellular RNA was extracted from human specimens (adjacent normal and tumor tissue), human GBM cells (U87MG and T98G), U87MG RR/TMZR and T98G RR/TMZR cells as described previously [24]. The primer sequences for respective genes is shown in Supplementary Table 1.

### Predicted transcription factor binding site in the upstream region of HSPB1

The ETS family of transcription factor has a high probability of regulating heat shock proteins [9, 11, 13]. Since our focus was HSPB1, we analyzed probable binding sites of all ETS family transcription factors in the upstream region of HSPB1. The analysis was done by using transcription factor prediction software such as ALIBABA, PATCH, MATCH, etc. Additionally, manual detection of probable sites was done by searching for established binding sites of ETS domains in the upstream region of HSPB1. This analysis indicated that the Fli-1 transcription factor has several binding sites in the upstream region of HSPB1. All the predicted binding sites were selected and primers (Supplementary Table 2) were designed for biological testing of these binding sites.

### Chromatin immunoprecipitation (ChIP) assays

ChIP assays using the SimpleChIP Enzymatic Chromatin IP kit, Cell Signaling Technologies protocol were done in order to confirm the binding of Fli-1 transcription factors to the upstream region of HSPB1. This was followed by immunoprecipitation using ChIP grade Fli-1 antibody. The entire procedure was followed as described previously [24].

### Confirmation of Fli-1 binding sites in the upstream region of HSPB1

In order to confirm the 3 binding sites obtained after ChIP assays, mutational analysis was done. This was done by mutating the predicted binding sites in order to destroy their binding ability. Oligos containing the mutations as well as control oligos without the mutations were synthesized. The length of the oligos was selected to be ~120-bp with the binding site in the middle, in order to ensure sufficient space for the binding of the transcription factor. This was followed by *in vitro* binding of the synthesized oligo and recombinant transcription factor Fli-1 in binding buffer. The DNA-protein was left overnight at 4°C to form a complex. This was subsequently used as the input for ChIP experiments done as described above.

### Soft agar assay

Anchorage independent growth assays in soft agar were used to monitor cellular transformation as described previously [24].

### Wound healing assay

The migration potential of GBM cells was analyzed by wound-healing assays or scratch assays as described previously [25].

### Chick chorioallantoic membrane (CAM) model of angiogenesis

CAM assays were performed to examine the angiogenic potential of GBM cells as described previously [5].

### Boyden chamber assays

Boyden chamber assays were carried out to check the invasive property of GBM cells as described previously [5].

### Western blotting analysis

Western blotting was performed to assess protein expression in human GBM, RR and TMZR cells as described previously [24].

### Transfection experiments

Transfection studies were performed to determine the effect of Fli-1 shRNA on T98G/ T98G RR/ T98G TMZR cells. Cells were grown in petri dishes and serum-starved overnight. The respective cells were transfected with Fli-1 shRNA/siRNA using a previously reported method [26].

### Cell proliferation assays

Antiproliferative effects of TMZ on SVGp12, U87MG, T98G, U87MG RR, U87MG TMZR, T98G RR and T98G TMZR cells were evaluated by MTT assays as described previously [27].

### Statistical analysis

Data presented as mean ± SEM unless otherwise stated. Statistical significance was determined by two-way analysis of variance (ANOVA) followed by a Student’s *t* test.

## SUPPLEMENTARY MATERIALS



## References

[1] Rajesh Y , Pal I , Banik P , Chakraborty S , Borkar SA , Dey G , Mukherjee A , Mandal M . Insights into molecular therapy of glioma: current challenges and next generation blueprint. Acta Pharmacol Sin. 2017; 38:591–613. 10.1038/aps.2016.167. 28317871 PMC5457688

[2] Das SK , Sarkar D , Cavenee WK , Emdad L , Fisher PB . Rethinking glioblastoma therapy: MDA 9/Syntenin targeted small molecule. ACS Chem Neurosci. 2019; 10:1121–1123. 10.1021/acschemneuro.9b00016. 30681320

[3] Kegelman TP , Das SK , Hu B , Bacolod MD , Fuller CE , Menezes ME , Emdad L , Dasgupta S , Baldwin AS , Bruce JN , Dent P , Pellecchia M , Sarkar D , et al. MDA-9/syntenin is a key regulator of glioma pathogenesis. Neuro Oncol. 2014; 16:50–61. 10.1093/neuonc/not157. 24305713 PMC3870820

[4] Rajesh Y , Biswas A , Mandal M . Glioma progression through the prism of heat shock protein mediated extracellular matrix remodeling and epithelial to mesenchymal transition. Exp Cell Res. 2017; 359:299–311. 10.1016/j.yexcr.2017.08.032. 28844885

[5] Rajesh Y , Banerjee A , Pal I , Biswas A , Das S , Dey KK , Kapoor N , Ghosh AK , Mitra P , Mandal M . Delineation of crosstalk between HSP27 and MMP-2/MMP-9: A synergistic therapeutic avenue for glioblastoma management. Biochim Biophys Acta Gen Subj. 2019; 1863:1196–1209. 10.1016/j.bbagen.2019.04.015. 31028823

[6] Yang C , Hong CS , Zhuang Z . Hypoxia and glioblastoma therapy. Aging (Albany NY). 2015; 7:523–524. 10.18632/aging.100795. 26298282 PMC4586094

[7] Hammerer-Lercher A , Mair J , Bonatti J , Watzka SB , Puschendorf B , Dirnhofer S . Hypoxia induces heat shock protein expression in human coronary artery bypass grafts. Cardiovasc Res. 2001; 50:115–124. 10.1016/S0008-6363(01)00198-5. 11282084

[8] Qiao N , Xu C , Zhu YX , Cao Y , Liu DC , Han X . Ets-1 as an early response gene against hypoxia-induced apoptosis in pancreatic β-cells. Cell Death Dis. 2015; 6:e1650. 10.1038/cddis.2015.8. 25695603 PMC4669796

[9] Foster CS , Dodson AR , Ambroisine L , Fisher G , Møller H , Clark J , Attard G , De-Bono J , Scardino P , Reuter VE , Cooper CS , Berney DM , Cuzick J . Hsp-27 expression at diagnosis predicts poor clinical outcome in prostate cancer independent of ETS-gene rearrangement. Br J Cancer. 2009; 101:1137–1144. 10.1038/sj.bjc.6605227. 19707199 PMC2768089

[10] Maroulakou IG , Bowe DB . Expression and function of Ets transcription factors in mammalian development: a regulatory network. Oncogene. 2000; 19:6432–6442. 10.1038/sj.onc.1204039. 11175359

[11] Oikawa T , Yamada T . Molecular biology of the Ets family of transcription factors. Gene. 2003; 303:11–34. 10.1016/S0378-1119(02)01156-3. 12559563

[12] Davidson B , Reich R , Goldberg I , Gotlieb WH , Kopolovic J , Berner A , Ben-Baruch G , Bryne M , Nesland JM . Ets-1 messenger RNA expression is a novel marker of poor survival in ovarian carcinoma. Clin Cancer Res. 2001; 7:551–557. 11297247

[13] Sizemore GM , Pitarresi JR , Balakrishnan S , Ostrowski MC . The ETS family of oncogenic transcription factors in solid tumours. Nat Rev Cancer. 2017; 17:337–351. 10.1038/nrc.2017.20. 28450705

[14] Ben-David Y , Bernstein A . Friend virus-induced erythroleukemia and the multistage nature of cancer. Cell. 1991; 66:831–834. 10.1016/0092-8674(91)90428-2. 1889087

[15] Oikawa T . ETS transcription factors: Possible targets for cancer therapy. Cancer Sci. 2004; 95:626–633. 10.1111/j.1349-7006.2004.tb03320.x. 15298723 PMC11159856

[16] Nazir SU , Kumar R , Singh A , Khan A , Tanwar P , Tripathi R , Mehrotra R , Hussain S . Breast cancer invasion and progression by MMP-9 through Ets-1 transcription factor. Gene. 2019; 711:143952. 10.1016/j.gene.2019.143952. 31265880

[17] Liu F , Walmsley M , Rodaway A , Patient R . Fli1 Acts at the top of the transcriptional network driving blood and endothelial development. Curr Biol. 2008; 18:1234–1240. 10.1016/j.cub.2008.07.048. 18718762

[18] Spyropoulos DD , Pharr PN , Lavenburg KR , Jackers P , Papas TS , Ogawa M , Watson DK . Hemorrhage, impaired hematopoiesis, and lethality in mouse embryos carrying a targeted disruption of the Fli1 transcription factor. Mol Cell Biol. 2000; 20:5643–5652. 10.1128/MCB.20.15.5643-5652.2000. 10891501 PMC86032

[19] Torlakovic EE , Slipicevic A , Flørenes VA , Chibbar R , DeCoteau JF , Bilalovic N . Fli-1 expression in malignant melanoma. Histol Histopathol. 2008; 23:1309–1314. 10.14670/HH-23.1309. 18785112

[20] Song W , Hu L , Li W , Wang G , Li Y , Yan L , Li A , Cui J . Oncogenic Fli-1 is a potential prognostic marker for the progression of epithelial ovarian cancer. BMC Cancer. 2014; 14:424. 10.1186/1471-2407-14-424. 24923303 PMC4089852

[21] Song W , Zhang T , Li W , Mu R , Zhang L , Li Y , Jin B , Wang N , Li A , Cui J . Overexpression of Fli-1 is associated with adverse prognosis of endometrial cancer. Cancer Invest. 2015; 33:469–475. 10.3109/07357907.2015.1069831. 26305602

[22] Scheiber MN , Watson PM , Rumboldt T , Stanley C , Wilson RC , Findlay VJ , Anderson PE , Watson DK . FLI1 Expression is correlated with breast cancer cellular growth, migration, and invasion and altered gene expression. Neoplasia. 2014; 16:801–813. 10.1016/j.neo.2014.08.007. 25379017 PMC4212256

[23] Liang X , Shi D , Yun J , Mao Y , Ouyang P , Su Z , Fu J , Hou J , Deng W , Xie F . Friend leukemia virus integration 1 expression has prognostic significance in nasopharyngeal carcinoma. Transl Oncol. 2014; 7:493–502. 10.1016/j.tranon.2014.04.015. 25171891 PMC4202802

[24] Rajesh Y , Biswas A , Kumar U , Das S , Banerjee I , Banik P , Bharti R , Nayak S , Ghosh SK , Mandal M . Targeting NFE2L2, a transcription factor upstream of MMP-2: A potential therapeutic strategy for temozolomide resistant glioblastoma. Biochem Pharmacol. 2019; 164:1–16. 10.1016/j.bcp.2019.03.025. 30885764

[25] Dey G , Bharti R , Ojha PK , Pal I , Rajesh Y , Banerjee I , Banik P , Parida S , Parekh A , Sen R , Mandal M . Therapeutic implication of ‘Iturin A’ for targeting MD-2/TLR4 complex to overcome angiogenesis and invasion. Cell Signal. 2017; 35:24–36. 10.1016/j.cellsig.2017.03.017. 28347875

[26] Dey G , Bharti R , Dhanarajan G , Das S , Dey KK , Kumar BN , Sen R , Mandal M . Marine lipopeptide Iturin A inhibits Akt mediated GSK3β and FoxO3a signaling and triggers apoptosis in breast cancer. Sci Rep. 2015; 5:10316. 10.1038/srep10316. 25974307 PMC4431395

[27] Parida S , Maiti C , Rajesh Y , Dey KK , Pal I , Parekh A , Patra R , Dhara D , Dutta PK , Mandal M . Gold nanorod embedded reduction responsive block copolymer micelle-triggered drug delivery combined with photothermal ablation for targeted cancer therapy. Biochim Biophys Acta Gen Subj. 2017; 1861:3039–3052. 10.1016/j.bbagen.2016.10.004. 27721046

