# Supplementary material for: Transcriptional regulation of HSPB1 by Friend leukemia integration-1 factor modulates radiation and temozolomide resistance in glioblastoma
Source: Oncotarget. 2020 Mar 31;11(13):1097–108. doi: 10.18632/oncotarget.27425 (PMC7138161; doi:10.18632/oncotarget.27425)
Supplement: Supplementary file 1 [file oncotarget-11-1097-s001.pdf]

Transcriptional regulation of HSPB1 by Friend leukemia integration-1 factor modulates radiation and temozolomide resistance in glioblastoma

SUPPLEMENTARY MATERIALS

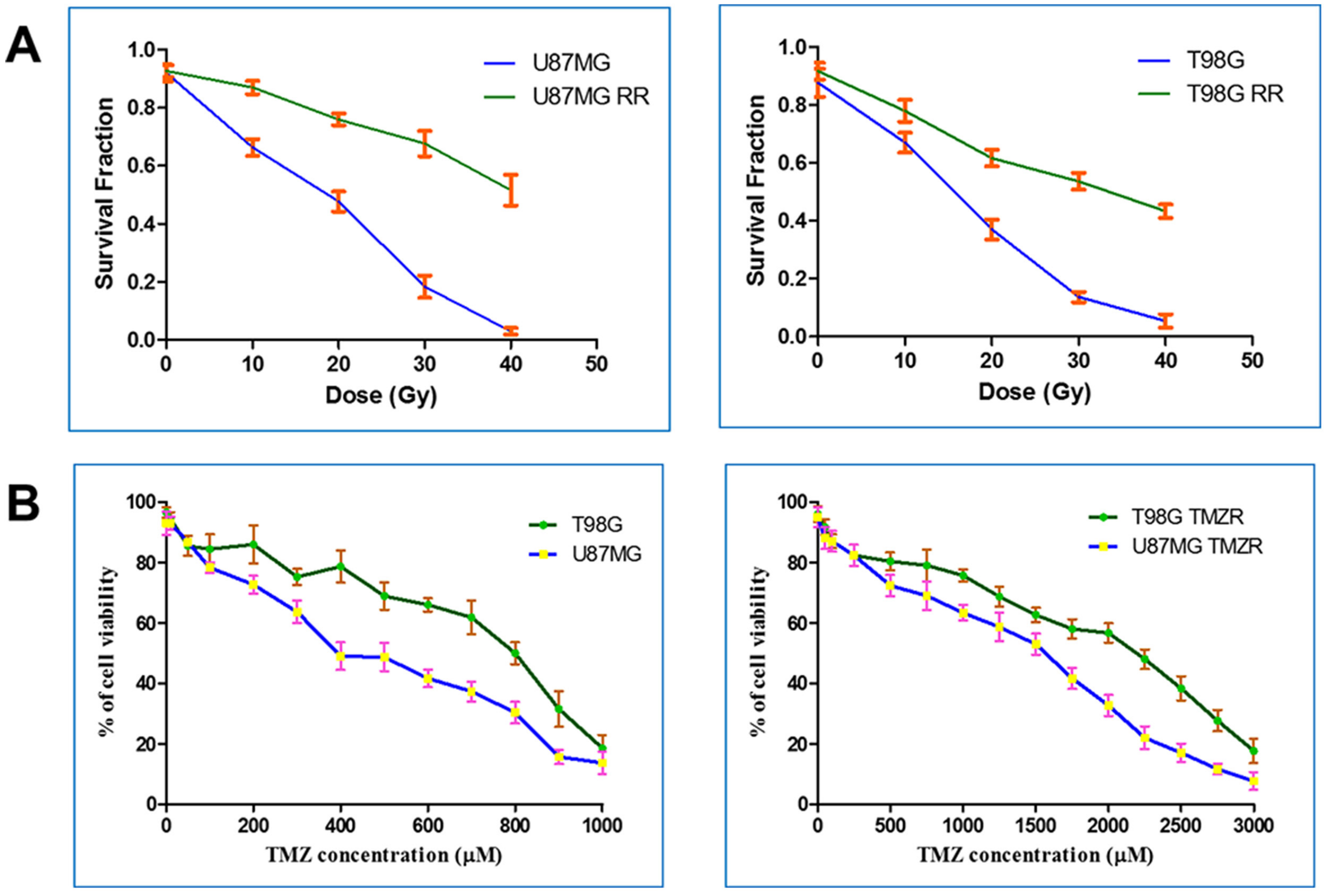

Supplementary Figure 1: (**A**) Determination of survival fraction in parental U87MG and T98G cells and radio-resistant U87MG RR and T98G RR cells. (**B**) Determination of IC50 of temozolomide (TMZ) in parental U87MG and T98G cells and TMZ-resistant U87MG TMZR and T98G TMZR cells.

GAGCTTGTGTCTACAAAGGGCTGGCAGCTCCCATCCAGAGCATGGCTGGGCCTGATAGAGCCACACCTGGAAGCATTTATAACCACACCTGACAGCTCCCTCTGGGGTGGG
GCACCCCCAGCTCCCGTCTGGGCCAAGGTGAGCAGGCAGAGGCAGATGAGGGCCCTCCCTGGCTCCTGACCTCACCTCGACTTTATACAGCTGGGGATGGGTGGGGCTG
GGAGCACTGGGGAGCAAAAACCTGGGGCTTTCTGATGCTGTCCCATGGGGTGTCAACCAGGAGCTCCCAAATGAGACCCACTGGGCTCATGACAATGACCCAGCCAGGGA
GGTGAGGTAGAAGGCAGGGGTCCTGTCTGCCTCCCACCATTGGCTCCCGAGAGCCCCAGGAGTTCAGAGGCCTTTCCAGGAGTGATCTCAGATTCATGGTCCAGCCCCAG
CTCTTCCCTGATGGGGAGCAGGTGGGTAAGACAGAAAGGCATCTAGGGCCGGTGGCTCACACCTGTAATCCCAGCACTCTGGGAGGCGAGGCAGGTGGATCACGAGGCC
AGGAGATCAAGACCATCCTGACTAACACAGTGAAACCCTGTCTCTACTAAAAAAAAAAAAAAAAACAATACAAAAAATTAGCCGGGCGTGGTGGTGGGCGCCTGTAGTCCCAG
CTACTCTGGAGGCTGAGGCAGGAGAATGGCGTGAACCCGGGAGGCGGAGCTTGCAGTGAGCCAAGATCGCACCACTGCACTCCAGCCTGGGTGACAGAGTGAGACTCCG
TCTCAAAAAAAAAAAGAAAGAAAGAAAGGCATCTAGGTCGGGCGCAGTGGCTCACGCCTGTGATCCCAGTGCTTTGGGAGGCCTAGGCAGAAAGATCTCTTGGGGCCAGG
AGTTCAAGACTAGTCTGGGCAACATGGTGCGACACCTGTCTCTACAAAAAATTTAAAAATTAGCCAAGTGTGGTGGCACACATCTGTAGTCCCAGTGACTCAGGAGGCTGAA
GCAGAAGAATCACTTGAGCCCAGGAGGTCAAGACTGCAATGAGCTATGATCACTCCACTGCACTCCAGCCTGGGCAACACAGCGAGACTCTGTCTCAACAAAAAAAAAAAAA
AAAAAGGAGAGAGAGAAAGGCATTTATGAGCTCTCTCTCAAAAAGGAAACTGAGGCTCAGAGAAGGCATGAGGACTTGCTGCTTCCCCAGCTCATCAAGGTGAGTGGCCTG
GAGTCTGCCCCGATTCTGGGCCTGGGCAGGGCAAGACTTCAGTGGCCTTGGCTCCCCATAGAAGTAGGAGGAGTGGAGCCTCAGTCCCAGGATAGGGACAGCAAGTACA
GTGGGACAGAAGAAGTGGGAGGCCCTGGGTACTAGAGAGGCAGGAGGTTGGCTCTGAGAGCACTTTACCGGAGACAGCAGAAAGCCATTTAATCTCTGTGCACAGCAGTT
TCCTTGTCTAAAAACCAGGGATAACAGGGCCGGGTGCGGTGGCTCACACCTGTAATCCCAGCACTTTGGGAGGCCGAGACAGGTGGCTGACTTGAGGTCAGGAGTTTGAG
ACCAACATGGCGAAACCCTGTTTCTACTAAAAATACAAAAATTAGCCGGGTGTGGTGGCTCATGCCTGTAATCCCAGCACTTTGGGAGGCCAAGGCAGGCGGCTCACTTGAG
GTCAGGAGTTTGAGACCAACATGGCGAAACCCCGTTTCTACTAAAAATACAGAAATTAGCCGGGTATGGTGGCACATGCCTGTAATCCCAGCCACTTGGGAGTCTGAGGCAG
GAGGATCGCTTGAACCCAGGAGGCGGAGGTTGCAGTGAGCTGAGATTGCGCCACTGCACTCCAGCCTGAGTGATAGAGTGAGACTCTGGCTCAAAAAAAAAAAAAAAAAA
AGACAAAAAAAAAAAAAACAACAACAACTAAAACAGGGACCTAAGTCAGGGGAGAAGGAAGGCTGGAATGGCCATGCCTACAAAGACAGGAAAGAGGGAATGTCAGGGTTG
TGGCAGGAGCCTAGGGCAGCACCCTCCCTCCAAGCTGGGAGATAGGAGCCAGGATCCCATTGGTGTTTAGATGGATGAGGTAGGGTCAAGGGGCAAGCTCCAGGCTCTAA
GCACTTCTGTGCAGACTCTACTACTTGGAGCTCCCCTCTCCCTCTGTGATGTGGGCATCAAAATCTGCAGAAAGGGAAACTGAGGCCTAGAGAAAGGAGGGGATTTGCCCA
GAATGACACAGGAAGTCCAGACTAATTCAAGTTTGCGGCATTTGAGGTTCCTGGCCAGTGTTTCTTTTTCTTTTTTGTTTTTTCTTTTTTGTTTGAGACAGAGTCTCGTTCTGTC
ACCGAGGCTGGAGTGCAGTGGCGCGATCTCAGCTCCCCGCAACCTCTGCCTCCTGGAATGATCCTCGTGCCTCAGCTTCCCCAGTAGCCGGGATTACAGGCACCCATCAC
ACCTGGCTAATTTTTTGTATTTTTGGCAGACACGGGGTTTCGGCATGTTGGCCAGGCTGGTCTTGAACTCCTGAACCCAGGCAATAAGTCTGCCTCAGACTCTCAAAGTGCT
GGGATTACAGGTGGGAGCCACCACGCCCGGCCAACCAGTGCTTCTTAAGGAAAGATACATATAGTAGGAGATGTGACAGTAATCTTCCTCCTGGCCCCTCTCAGCACTGTGC
CATGGAGGTTCTGATCTGGGCCAGCTGCGCTGTTAGGGGAAGGGGGAAAATGGCCAAACCCCATGTATTCTCCTTCCTTTTCCCTCTCTTCAATCCCAGGCAGGTAACAATA
ATTATGATGTACCAGGCTCCGGGCCAAAGTCATCACCTGCATTAGGTCACCAAATCCTCAGGCCAACCCATTACAGTCATGAGTCACTTAATAACAGACACATTCAACAAATTC
CTTGTTAGGCAATTTCATCATTGTGGTAACATCTCAGAGTGGACTTACACAAACCTAGATGGTCTAGCCTACTACACACCTAAGCCATATGGTCTAGCCTACTGCTCCTAGGCTA
CAAACCTCTACAGCATATGACTGCACCAAATATTGAAGGCAGCTGCAACATGAGGGTAAGTATTAGTGTTTCTAAAGATAGAAGATGGCCAGGCGCGGTGGCTCACGCCTGTA
ATCCTAGCACTTTGGGAGGCCCAGGCGGGTGGATTACTTGAGGTCAGGAGTTCAAGACCAGCCTGGCGAACATGGTGAAACCCCATCTGTACTAAAAATACAAAAATTAGCC
AGTGTCGCGGCACTTGCCTGTAGTCCCAGCTACTCAGGAGGCTGAGGCAGAAGAATCACTTGAACCTGGGAGGTAGAGGTTGGAGTGAGCCTAGATCAGGCCACTGCACT
CCAGCCTGGGCGACAGAGGGAGACTCCATCTCAAAATAAATAAATAAATAAATAAATAAAACATAGAAGATGTACAGTAAAAACACGGTAATTGTTTTTGTTTGTTTGTTTTGAGA
CAGGGTCTTGTTCTGTCATGCGGACTGGAGTGCAGTGGCACCATCAGGCTCACTGCAGCCTCGACCTCCTTGGCTCAAGTGCTCCTCCCACCTCAGCCTCCTGAGTATCTG
GGACTACAGGTCCACGCCACCATGCCTGGCTAATTTGTTCTGAATTTTAGTAGAGATGGGGTCTCACTGTGTTGCCCAGGCTTGTTCCAGCCTTCTGGCTTCAAGTAATCCTC
CCACATCAGCCTCCCAAAGTGCTAAGATTACAGATGTCAGCCACTGCACCCAGCCAGTAATATAATTTTATGGGACCACCTTCATATTTGCTGTCCCTTGCTGACTTACACATCT
TTATGCAATGCATGACTGTTACCATCATTATCATCTCTATTTTCCAGATGGGGAAACTGAGGCACAAAGAATCTAACTTGCACAAGTTCATCTGCTTAGTGATGGAACAAAGATG
TGAATTCAGGCAGTCTGGCTTCAAAGTCCACACGCCTAACAACCACACCAGATTACTAGATTGCTTTTTTCTTTTTTCTTTTTTTTTTTTTGAGATGGAGTCTCACTCTGTCACC
CAGGCTGGAGTACAGTGGTGAGATCTCGGCTCACTGCAACCTCTGCCTTCTGGGTTCAAGCAATTCTCCTGCCTCAGCCTCCCCAGCAGCTGCGATTACAGGCGCCCGCCA
CCACACCCAGCTAATTTTTGTATTTTTAGTAGAGATGGGGTTTCACCATGTTGGCCAGGCTGGTCTCAAACTCCTGACCTCTGGTGATCCTCCCACCTCGGTCTCCCAAAGTG
CTGGGATTACAGGCGTGAGCCACCACGCCCAGCCCAGACTGCTTTATTTTTGTATTTGTATTTATTCATTTACTTATTTTGAGACAGGGTTTTGCTCTGTAGCCCAGGCTGAAGT
GCAGTGGTGCAATCCAGCTCACCACAGCCTCTACTCACCGGGGTTCAAAGGATCCTCCTGCTTCAGCCTCTGGAGTAGCTGGGGCCACAGGCATGCACCACCATGCCCAG
CTAATTTTTAAATATTTTTTGGTAGAAGTAGGGTCTCACTATGTTGCCCAGACTGGTCTCAAACTCCTAGCCTCAAGGGACCCTTCTGCCTTGGCCTCCCAAAGTGCTGAGATT
ACAGGCATGAGCCATGCACCCAGCCCCTTTTTAAAATTTTTTTGAGAGACAAGACTTTGATCTGTTGCCTAGGCTGGAGTGCAGTGGTGAGATCATAGCTCACTGCAGCCTCA
ACTCCTGGGCTCAAGCACCAGACTCCTTTTATCACATTCTATCTCACACGCGTGTGGTTCCAATCCTGCCTCTGCCACTTCTCAGTTGTATGCCCCAACCCAACCTGTCTGGC
TCTGTCCTCCTTAACAGAAGGACGGCCCTGGCCACGGGCCACAGCCAGCAACGCTTAAGCACCAGGGCCGGCGAGTGCCCTGCCGTGGCACGGCTCCAGCGTCGCGCT
CTCGAATTCATTTGCTTTCCTTAACGAGAGAAGGTTCCAGATGAGGGCTGAACCCTCTTCGCCCCGCCCACGGCCCCTGAAC

Turquoise Region: Primers (Forward and Reverse)
Yellow Region: Amplified region
Other highlights: Predicted binding regions

Supplementary Figure 2: Sequence of HSPB1 5 kb upstream region for prediction of transcription factor binding sites.

Identifier	Position	Mismatches	Score	Binding Factor	Sequence
AS$ETS1_10	2219 (+)	0	100.00	c-Ets-1	ACAGGAAGTC
ELK1$CONS_02	2219 (+)	0	90.91	Elk-1	ACMGGAAGTNC
AS$CETS1P54_07	2219 (+)	0	100.00	c-Ets-1 54	ACAGGAAGTC
TCF2ALPHA$CONS	2220 (+)	0	87.50	TCF-2alpha	SAGGAAGY
FLI1$CONS_01	2220 (+)	0	93.75	Fli-1	CMGGAAGT
MOUSE$TIMP1_02	2220 (+)	0	100.00	c-Ets-1	CAGGAAG
AS$ELK1_13	2220 (-)	0	100.00	Elk-1, SAP-1a, SAP-1b, SRF	TTCCTG
PEA3$CONS	2221 (+)	0	91.67	c-Ets-1 54, c-Ets-1 68, c-Ets-2 58-64,	AGGAAR

```
==================================================================================
seq( 2220.. 2279)     aggaagtccagactaattcaagtttgcggcatttgaggttcctggccagtgtttcttttt
Segments:
1.1.3.0     2212 2221 p=
3.1.1.9     2217 2226 =K-2a==
3.5.2.0     2217 2226 Ets-1_=
1.6.1.0     2259 2268                                        =AP-2alph=
1.3.1.2     2260 2269                                         ====USF===
9.9.539     2261 2270                                          ====NF-1==
9.9.428     2270 2279                                                   ===ISGF-3=
3.1.1.2     2272 2281                                                     ====Antp
1.1.1.5     2273 2282                                                      ====GCN
==================================================================================
```

```
==================================================================================
seq( 1740.. 1799)     atcccagccacttgggagtctgaggcaggaggatcgcttgaacccaggaggcggaggttg
Segments:
9.9.539     1732 1741 ==
1.1.3.0     1738 1747 /EBPalp=
1.1.1.1     1758 1767                   ===c-Jun==
1.1.1.2     1759 1768                    ===c-Fos==
9.9.29      1760 1769                     ====AP-1==
1.2.1.0     1762 1771                       =====E1===
1.2.2.0     1762 1771                       ====MyoD==
3.5.2.0     1762 1771                       =c-Ets-1_=
2.3.1.0     1786 1796                                               =====Sp1===
2.2.1.1     1797 1806                                                          ===
==================================================================================
```

```
==================================================================================
seq( 4800.. 4859)     cctgtctggctctgtcctccttaacagaaggacggccctggccacgggccacagccagca
Segments:
2.2.1.1     4817 4826                  ===GATA-1=
3.5.2.0     4817 4826                  ==c-Ets-1=
2.3.1.0     4828 4841                             ======Sp1=====
2.3.1.0     4835 4848                                    ======Sp1=====
1.3.1.2     4836 4845                                     ====USF===
1.3.2.1     4836 4845                                     ===c-Myc==
1.3.2.2     4836 4845                                     ====Max1==
1.6.1.0     4836 4845                                     =AP-2alph=
9.9.539     4838 4847                                       ====NF-1==
2.3.1.0     4847 4856                                                ====Sp1===
1.1.1.1     4852 4861                                                     ===c-Jun
1.5.1       4852 4861                                                     ====EF-C
==================================================================================
```

Supplementary Figure 3: Prediction of Fli-1 transcription factor in 5 KB upstream region of HSPB1 using ALIBABA and PATCH detection software.

Binding Site 3:
TTAGCCGGGTATGGTGGCACATGCCTGTAATCCCAGCCACTTGGGAGTCTGAGGCAGGAGGATCGCTTGAACCCAGGAGGCGGAGGTTGCAGTGAGCTGAGATTGCGCCA
Mutation:
TTAGCCGGGTATGGTGGCACATGCCTGTAATCCCAGCCACTTGGGAGTCTTGTAAGTACAGATCGCTTGAACCCAGGAGGCGGAGGTTGCAGTGAGCTGAGATTGCGCCA
Binding Site 6:
AGGCTGGAGTGCAGTGGCGCGATCTCAGCTCCCCGCAACCTCTGCCTCCTGGAATGATCCTCGTGCCTCAGCTTCCCCAGTAGCCGGGATTACAGGCACCCATC
Mutation:
AGGCTGGAGTGCAGTGGCGCGATCTCAGCTCCCCGCAACCTCTGCCTCCTTACGTGATCCTCGTGCCTCAGCTTCCCCAGTAGCCGGGATTACAGGCACCCATC
Binding Site 7:
TGCTTCTTAAGGAAAGATACATATAGTAGGAGATGTGACAGTAATCTTCCTCCTGGCCCCTCTCAGCACTGTGCCATGGAGGTTCTGATCTGGGCCAGCTGCGCTGTTAGGGGAAGGGGGAAAATGGCCAAA
Mutation:
TGCTTCTTAATACGAGATACATATAGTAGGAGATGTGACAGTAATCTTCCTCCTGGCCCCTCTCAGCACTGTGCCATGGAGGTTCTGATCTGGGCCAGCTGCGCTGTTAGGGGAAGGGTACGAATGGCCAAA

Supplementary Figure 4: Sequence of ~120-bp upstream region of HSPB1 containing the mutated binding sites 3, 6 and 7 and the respective control oligonucleotides without mutations.

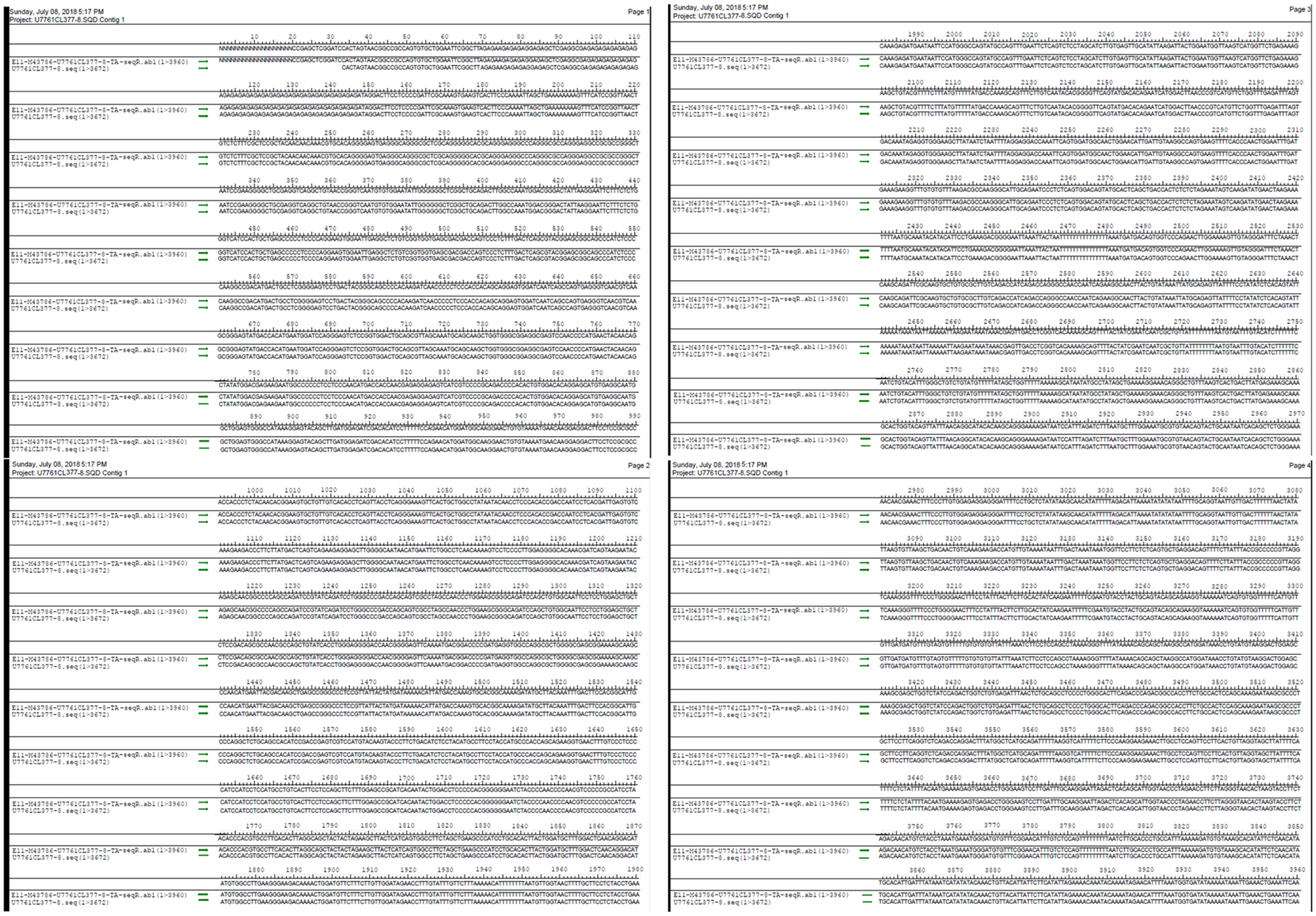

Supplementary Figure 5: The sequencing data of the overexpressing Fli-1 plasmid.

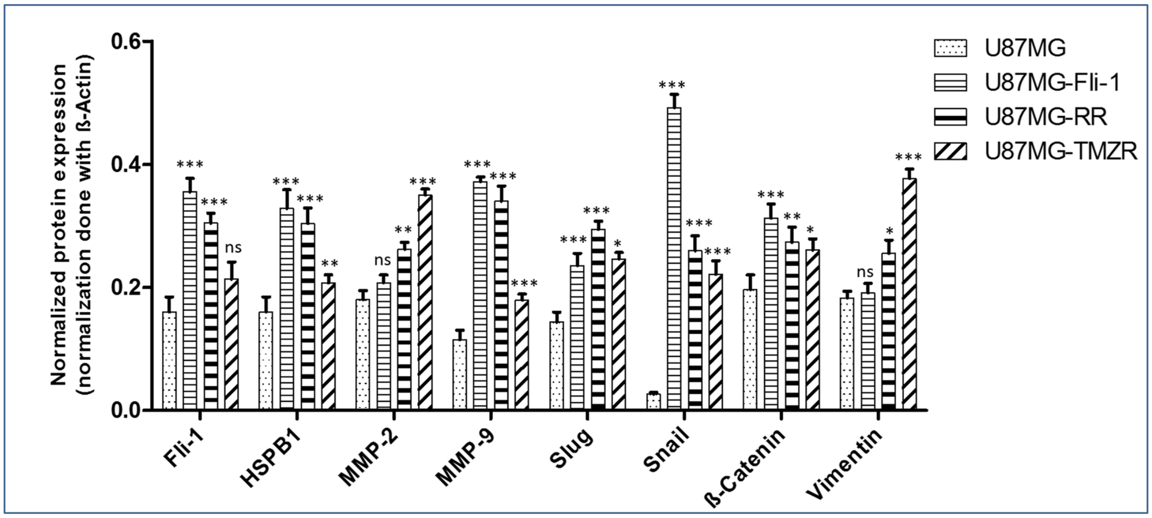

Supplementary Figure 6: Densitometry plot of Figure 5C. Western blotting analysis of Fli-1, HSPB1, MMP-2, MMP-9 and EMT proteins in U87MG, U87MG RR, TMZ-resistant U87MG and U87MG Fli-1 cells.

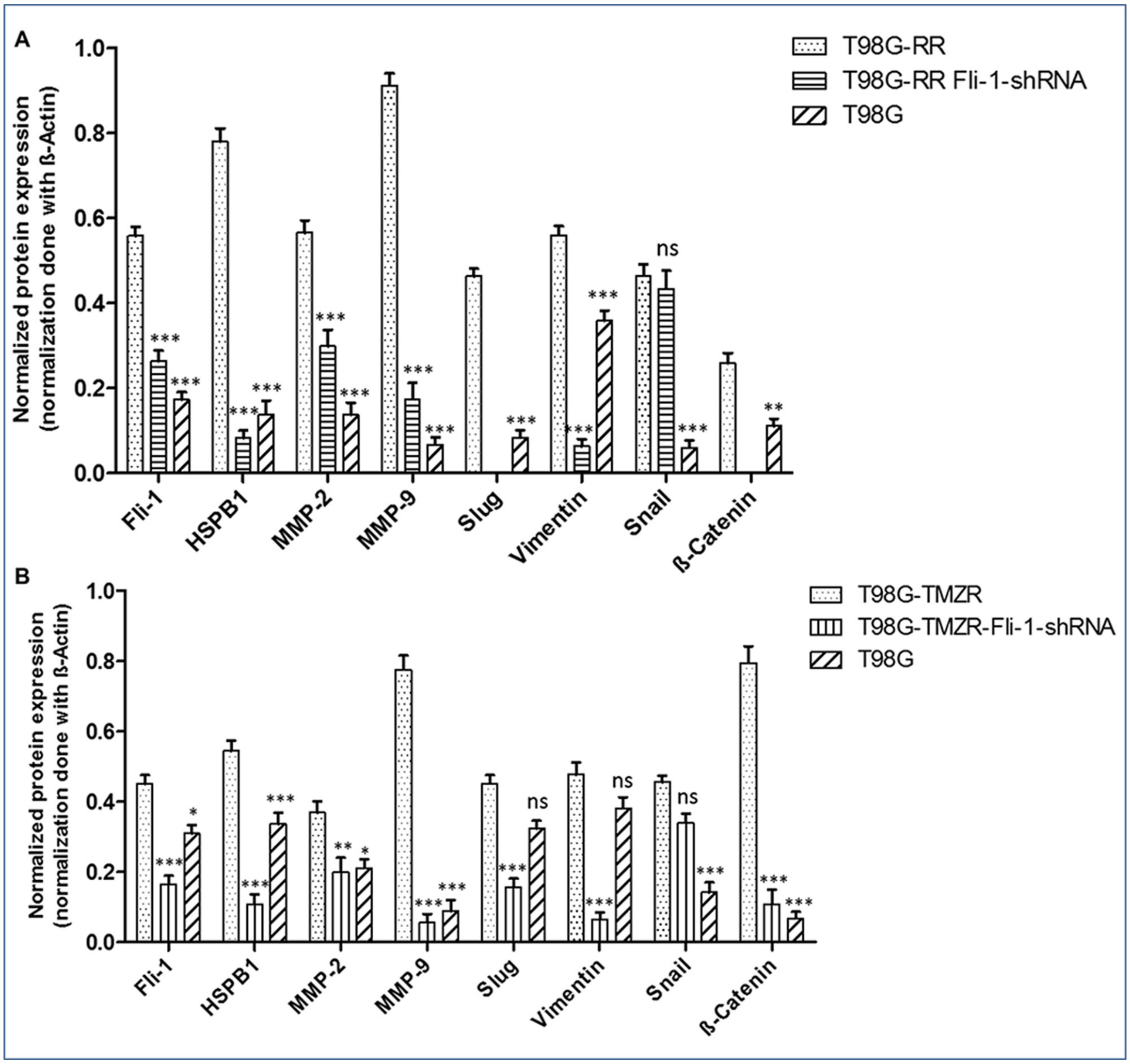

Supplementary Figure 7: (**A**, **B**) Densitometry plot of Figure 6E and 6F. Western blotting analysis of Fli-1, HSPB1, MMP-2, MMP-9 and EMT proteins in parental T98G, radio-resistant T98G RR, TMZ-resistant T98G, T98G RR Fli-1 shRNA and T98G TMZR Fli-1 shRNA cells. Each bar represents the average of three independent experiments. The level of significance is indicated by $^{*}P < 0.05$, $^{**}P < 0.01$, $^{***}P < 0.001$.

Supplementary Table 1: The primer sequences for qRT-PCR

	Primer Sequence (5′–3′)
**HSPB1**	**F** GAGACTGCCGCCAAGTAAAG<br>**R** TTTGACAGGTGGTTGCTTTG
**HSP 40**	**F** GGAAGGGACCCATTTTCATT<br>**R** CCACTTCCAAAAGACGGAAA
**HSP 70**	**F** CCAGCTGAAGAAGGGTCAAG<br>**R** CAGGATCTGGATAGGGCAAA
**HSP 90**	**F** AGACCCAGTCTTGTGGATGG<br>**R** ACTCCCCTTTCCCCCTAAAT
**β-Catenin**	**F** TGCAGTTCGCCTTCACTATG<br>**R** ACTAGTCGTGGAATGGCACC
**Slug**	**F** TGTGTGGACTACCGCTGC<br>**R** TCCGGAAAGAGGAGAGAGG
**Snail**	**F** TGCAGGACTCTAATCCAGAGTTTACC<br>**R** GTGGGATGGCTGCCAGC
**Vimentin**	**F** TGTCCAAATCGATGTGGATGTTTC<br>**R** TTGTACCATTCTTCTGCCTCCTG

Supplementary Table 2: The ChIP primer sequences for Fli-1 binding sites in the 5 kb upstream of HSPB1

Binding Site	Primer Sequence (5′–3′)
1	Forward: GTGTCTACAAAGGGCTGGCA<br>Reverse: CTGCTCACCTTGGCCCAG
2	Forward: GGAGAGAGAGAAAGGCATTTATGA<br>Reverse: CCACTGAAGTCTTGCCCTG
3	Forward: CGAAACCCCGTTTCTACTAAAA<br>Reverse: CTCACTCTATCACTCAGGCTGG
4	Forward: CAACAACTAAAACAGGGACCTAAGTC<br>Reverse: GGCTCCTATCTCCCAGCTTG
5	Forward: TCTCCCTCTGTGATGTGGG<br>Reverse: GAAACACTGGCCAGGAACC
6	Forward: GAGACAGAGTCTCGTTCTGTCAC<br>Reverse: TACAAAAAATTAGCCAGGTGTGAT
7	Forward: AACCAGTGCTTCTTAAGGAAAGATAC<br>Reverse: CCTGGGATTGAAGAGAGGG
8	Forward: TGCAATGCATGACTGTTACCA<br>Reverse: TAGGCGTGTGGACTTTGAA
9	Forward: TCACACGCGTGTGGT<br>Reverse: TGGAGCCGTGCCACG
